# *Ab Initio* Molecular Dynamics Study of Methanol-Water Mixtures under External Electric Fields

**DOI:** 10.3390/molecules25153371

**Published:** 2020-07-24

**Authors:** Giuseppe Cassone, Adriano Sofia, Jiri Sponer, A. Marco Saitta, Franz Saija

**Affiliations:** 1 CNR-IPCF, Viale F. Stagno d’Alcontres 37, 98158 Messina, Italy; 2 Dipartimento di Matematica “G. Peano”, Università degli Studi di Torino, Via Carlo Alberto 10, 10123 Torino, Italy; adriano.sofia@unito.it; 3 Institute of Biophysics of the Czech Academy of Sciences, Královopolská 135, 61265 Brno, Czech Republic; sponer@ncbr.muni.cz; 4 Sorbonne Université, Msuéum National d’Histoire Naturelle, UMR CNRS 7590, IMPMC, F-075005 Paris, France; marco.saitta@sorbonne-universite.fr

**Keywords:** *ab initio* molecular dynamics, electric fields, methanol, aqueous solutions, proton transfer, chemical reactivity

## Abstract

Intense electric fields applied on H-bonded systems are able to induce molecular dissociations, proton transfers, and complex chemical reactions. Nevertheless, the effects induced in heterogeneous molecular systems such as methanol-water mixtures are still elusive. Here we report on a series of state-of-the-art *ab initio* molecular dynamics simulations of liquid methanol-water mixtures at different molar ratios exposed to static electric fields. If, on the one hand, the presence of water increases the proton conductivity of methanol-water mixtures, on the other, it hinders the typical enhancement of the chemical reactivity induced by electric fields. In particular, a sudden increase of the protonic conductivity is recorded when the amount of water exceeds that of methanol in the mixtures, suggesting that important structural changes of the H-bond network occur. By contrast, the field-induced multifaceted chemistry leading to the synthesis of e.g., hydrogen, dimethyl ether, formaldehyde, and methane observed in neat methanol, in 75:25, and equimolar methanol-water mixtures, completely disappears in samples containing an excess of water and in pure water. The presence of water strongly inhibits the chemical reactivity of methanol.

## 1. Introduction

Alcohol-water mixtures have extensively been investigated due to their academic and industrial relevance [1]. In the literature, investigations on these solutions were particularly focused on the role of the hydrophobic headgroups of alcohols in determining the physical properties of water and vice versa [2]. Owing to its own intrinsic molecular simplicity, one of the most studied systems is represented by methanol-water mixtures. Methanol (CH${}_{3}$OH)—the simplest alcohol—can classically be considered as a water molecule where a hydrogen-donor site is replaced by a hydrophobic methyl group. Such a substitution produces several differences between these two H-bonded liquids. In fact, the presence of a methyl group instead of a hydrogen atom in the methanol molecule induces considerable effects in the water and methanol clustering properties, not only when mixed but also in neat samples. From a topological point of view, water exhibits its peculiar tetrahedrally-coordinated structure, whereas methanol contains linear and irregular chains of H-bonded molecules(the interested reader may refer, e.g., to Figure 1 of Ref. [3]). The methanol hydroxyl (OH) group allows alcohols to form H-bonds and it is responsible for their good solubility in aqueous environments. By contrast, the hydrophobic methyl (CH${}_{3}$) group does not participate in the H-bond network, neither in pure methanol nor when the simplest alcohol is solvated in water. Because of such an amphiphilic nature, the microscopic structures of hydrated methanol in liquid water have attracted noticeable attention. Several experimental approaches have shown that the anomalous thermodynamics of alcohol-water systems arises mainly from the incomplete mixing at the molecular level, which exhibits partially segregated microstructures, both into water-rich and alcohol-rich components depending on the relative concentrations [4,5,6,7,8,9,10,11,12]. In order to supplement or corroborate the experimental results, many computer simulation studies have been performed at different levels of sophistication. Classical force-fields molecular dynamics (MD) and Monte Carlo methods have been employed to investigate the dynamical and structural features of methanol-water mixtures, providing also interesting analysis of H-bonding effects [13,14,15,16,17,18,19,20,21,22]. Far less are the investigations based on *ab initio* molecular dynamics (AIMD) simulations [23,24,25]. Morrone et al. [26] analyzed the structural and proton transport properties of methanol-water mixtures at two different molar ratio regimes in which the mixture consisted of two bi-percolating H-bonded networks. They observed both enhanced structuring of water as the methanol mole fraction increases and revealed the existence of separate H-bonded water and methanol networks, in agreement with neutron diffraction experiments [4,6]. The application of static electric fields is able to modify the H-bond structure of neat methanol [27,28] and of pure water [29,30,31,32] leading to crucial implications in electrochemistry [33,34,35,36,37] and in biology [38]. Also for this reason, in a recent article, He et al. have investigated the structure of methanol-water mixtures showing that, at high methanol concentrations, the application of the field affects the structural evolution of the H-bond network, in that the field creates a “hollow channel” in the intermolecular interactions [39].

The aim of this study is to unveil the molecular mechanisms and chemical reaction networks of methanol-water mixtures at different molar ratios when intense static electric fields are applied. By using state-of-the-art AIMD simulations, we investigate the field-induced proton transfer phenomenon as a function of the water concentration. At large water concentrations, we note an abrupt change of the conductivity properties of methanol, which can be ascribed to a percolating effect of the H-bond network. Beyond the molecular dissociation threshold, our simulations reveal that strong electric fields can promote peculiar reaction channels able of triggering important chemical reactions. On the other hand, such a field-induced enhancement of the chemical reactivity is drastically hampered by the presence of water.

## 2. Materials and Methods

We used the software package CP2K [40,41], based on the Born-Oppenheimer approach, to perform AIMD simulations of five samples (i.e., neat methanol, 75:25 (X025), 50:50 (X050), 25:75 (X075) methanol-water mixtures, and neat water) under the action of static and homogeneous electric fields applied along a given direction (corresponding to the *z*-axis).AIMD simulations do not employ specific parametrizations for mimicking the interactions between atoms or molecules but explicitly solve the electronic problem within a rigorous Density Functional Theory (DFT) framework. This way, interactions between nuclei genuinely result from the forces emerging from the potential energy surfaces of the electronic subsystem. The implementation of an external field in numerical codes based on DFT can be achieved by exploiting the Modern Theory of Polarization and Berry’s phases [42,43,44] (see, e.g., Ref. [45]). Thanks to those seminal works, nowadays AIMD simulations under the effect of static electric fields with periodic boundary conditions are almost routinely carried out (see, e.g., [46]). The neat methanol sample contained 50 CH${}_{3}$OH molecules (i.e., 300 atoms) arranged in a cubic cell with edge equal to 14.99 Å, so as to reproduce the liquid methanol experimental density of 0.79 g·cm${}^{-3}$ at room temperature. The 75:25 methanol-water mixture sample was composed of 60 CH${}_{3}$OH and 20 H${}_{2}$O molecules (i.e., 420 atoms) placed in a cubic box with edge of 16.22 Å. The 50:50 (equimolar) methanol-water sample was composed of 40 CH${}_{3}$OH and 40 H${}_{2}$O molecules (i.e., 360 atoms) placed in a cubic box with edge equal to 15.51 Å. The 25:75 methanol-water mixture sample was composed of 20 CH${}_{3}$OH and 40 H${}_{2}$O molecules (i.e., 300 atoms) arranged in a cubic box having edge of 14.73 Å. Finally, the neat water sample was composed of 128 H${}_{2}$O molecules (i.e., 384 atoms) arranged in a cubic box with edge equal to 15.81 Å, so as to reproduce a density of 0.97 g·cm${}^{-3}$. As usual, in order to minimize nonphysical surface effects, all structures were replicated in space by employing periodic boundary conditions. The intensity of the electric field was gradually increased with a step increment of 0.05 V/Å from zero up to a maximum of 0.50 V/Å. In the zero-field cases we performed dynamics of 50 ps for each investigated sample whereas, for each other value of the field intensity, we ran dynamics of 10 ps, thus accumulating a global simulation time equal to 750 ps whereas a time-step of 0.5 fs has been chosen.

Wavefunctions of the atomic species have been expanded intriple-zeta valence plus polarization (TZVP) basis sets with Goedecker-Teter-Hutter pseudopotentials using the GPW method [47]. A plane-wave cutoff of 400 Ry has been imposed. Exchange and correlation (XC) effects were treated with the Becke-Lee-Yang-Parr (BLYP) [48,49] density functional. Moreover, in order to take into account dispersion interactions, we employed the dispersion-corrected version of BLYP (i.e., BLYP-D3) [50,51]. The adoption of the BLYP-D3 functional has been dictated by the widespread evidence that such a functional, when dispersion corrections are taken into account, offers one of the best adherence with the experimental results related to water among the standard GGA functionals [52,53]. It is well-known indeed that neglecting dispersion corrections leads to a severely over-structured liquid (see, e.g., Ref. [54] and references therein). In order to counteract the overstructuring of intermolecular interactions typically induced by GGA XC functionals, all methanol-containing simulations were executed at a temperature of 330 K whereas the neat water simulation was executed at 350 K. The dynamics of nuclei was simulated classically within a constant number, volume, and temperature (NVT) ensemble, using the Verlet algorithm whereas the canonical sampling has been executed by employing a canonical-sampling-through-velocity-rescaling thermostat [55] set with a time constant equal to 10 fs.

## 3. Results and Discussion

### 3.1. Field-Induced Proton Transfer

**Figure 1:**
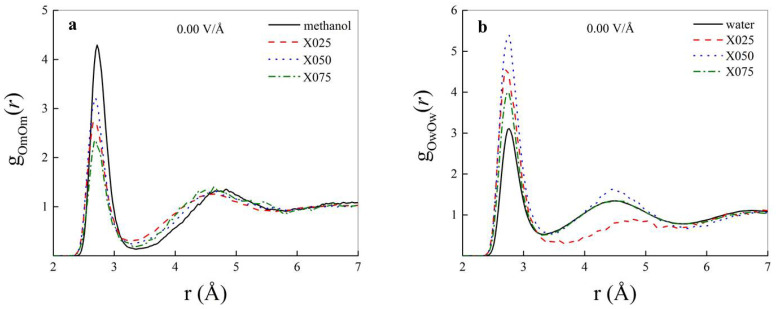
Methanol oxygen-methanol oxygen (O${}_{m}$O${}_{m}$) (**a**) and water oxygen-water oxygen (O${}_{w}$O${}_{w}$) (**b**) radial distribution functions (RDFs) in the zero-field regime in samples containing neat methanol (**a**) and neat water (**b**) (solid black curves), mixtures composed of 75:25 (dashed red curves), 50:50 (dotted blue curves), and 25:75 (dashed-dotted green curves) molar fractions of methanol and water molecules, respectively.

Atomistic radial distribution functions (RDFs) represent a useful tool capable to monitor the short-range positional order typical of H-bonded systems. Albeit in methanol-water mixtures several RDFs can be defined, the most relevant ones are the oxygen-oxygen (OO) RDFs, these latter being capable to trace local molecular interactions. As shown in Figure 1a, the inclusion of increasingly larger fractions of water molecules in liquid methanol induces a non-monotonic decrease of the first peak height of the methanol oxygen-methanol oxygen (O${}_{m}$O${}_{m}$) RDF. In fact, the height of the O${}_{m}$O${}_{m}$ RDF first maximum of a 50:50 methanol-water (X050) mixture is higher than those exhibited by the same RDF both in 75:25 (X025) and in 25:75 methanol-water (X075) mixtures. On the other hand, the location of the first peak is independent of the amount of solvated water, falling at a distance of ∼2.71 Å both in the neat methanol sample and in all the other methanol-water mixtures. Such a value corresponds exactly to that obtained by means of Car-Parrinello [56] MD simulations of bulk neat methanol [28] and it is in fairly good agreement with experimental data (∼2.74 Å) [57,58]. Besides, accordingly to the findings reported in investigations based on classical force-fields MD [17], inclusion of larger fractions of water molecules affects the second peak of the O${}_{m}$O${}_{m}$ RDF, as shown in Figure 1a. This implies that whereas water inclusion does not significantly perturb direct methanol-methanol interactions, it induces a slight compression of the second methanol solvation shell, as witnessed by the shift toward shorter distances of the second peak location.

When relatively small amounts (i.e., 25% molar fraction) of water are solvated in methanol, longer-range order of the aqueous subsystem is disrupted by the preponderant presence of methanol molecules, as shown by the flatness of the second peak of the water oxygen-water oxygen (O${}_{w}$O${}_{w}$) RDF in Figure 1b (dashed red curve). By contrast, a sudden structuring of the water system is recorded in equimolar 50:50 methanol-water mixtures. In fact, both the first and the second peak height exhibit values considerably larger than those typical of bulk neat water. This means that the presence of the hydrophobic methyl groups of methanol constrains the water subsystem into a more rigid intermolecular structure. However, such a steric confinement is recorded only for one specific ratio of the molar fractions of methanol and water molecules (i.e., 50:50). As shown in Figure 1b, indeed, when the amount of water is larger than the amount of methanol in a methanol-water mixture, the O${}_{w}$O${}_{w}$ RDF overlaps that recorded in pure water samples, with the exception of a reminiscent higher first peak (dashed-dotted green curve).

**Figure 2:**
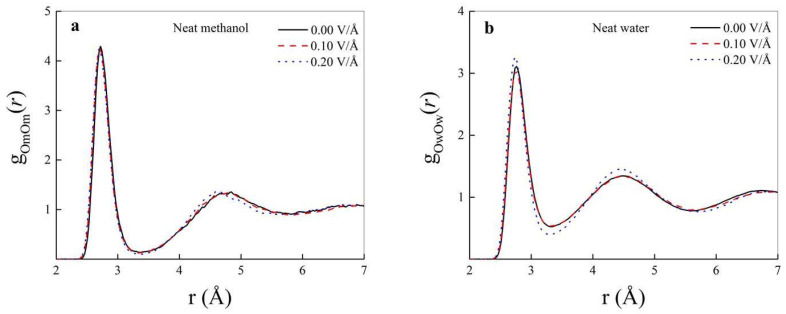
Oxygen-oxygen radial distribution functions (RDFs) in bulk neat methanol (**a**) and in bulk neat water (**b**) in the zero-field regime (solid black curves) and for different field strengths. Dashed red curves: 0.10 V/Å; dotted blue curves: 0.20 V/Å.

When a static and homogeneous electric field is switched on, polarization effects are induced [46]. Similarly to other H-bonded systems [59], when field strengths larger than 0.01 V/Å are applied on samples of liquid methanol or liquid water, a detectable fraction of molecules aligns toward the field direction [31,60]. The application of an external electrostatic potential gradient forces, indeed, dipole vectors to align along the field direction. Although a slight strengthening of the intermolecular interactions in neat methanol upon exposure to field intensities on the order of ∼0.10–0.20 V/Å has been recorded [28], no marked field-induced changes of the methanol structure are observed at these field regimes, as shown in Figure 2a. In fact, only a very feeble structuring of the second solvation shell can be appreciated in bulk neat methanol for fields as intense as 0.20 V/Å. The larger dipole moment and dielectric constant of water, along with a more percolated H-bond network, confer to a neat water sample a more pronounced structural response to external static electric fields. As shown in Figure 2b, indeed, a field strength of 0.20 V/Å sizably enhances the structuring of the H-bond interactions in bulk liquid water leading to higher (lower) peaks (dips) in the respective O${}_{w}$O${}_{w}$ RDF with respect to the zero-field case. As recently reported in an ab initio investigation of the spectroscopic features of liquid water under static electric fields, intensities on this order of magnitude are able to render the water structure more “ice-like”, in the sense that the arrangement of water molecules exhibits an increased positional and orientational order. [32]. In fact, the structuring highlighted by the RDF of Figure 2b represents also the mirror of a field-induced increase of the tetrahedral order typical of condensed aqueous phases [32].

**Figure 3:**
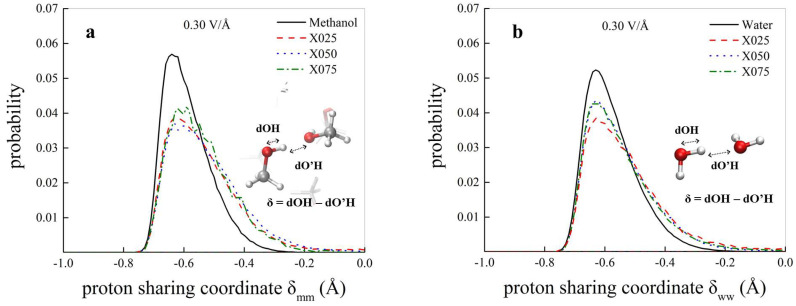
Probability distributions of the proton sharing coordinate δ between methanol molecules (**a**) and between water molecules (**b**) at 0.30 V/Å in neat samples (black curves) and in methanol-water mixtures at different molar fractions. In the insets, the definition of the coordinate, which is determined for every hydrogen atom in the systems involved in a tight H-bond, is shown.

It is well-established that static fields as intense as 0.30 V/Å are necessary in order to induce the phenomenon known as *protolysis* in liquid water [29,32,61,62,63]:

(1) $$2H_{2}O\overset{E}{\to }H_{3}O^{+}+OH^{-}\;,$$

which results in the concept of *p*H and where hydronium H${}_{3}$O${}^{+}$ and hydroxide OH${}^{-}$ ions are produced. Similar field intensities (0.30 V/Å) are also required in order to induce molecular dissociations in neat methanol [28], as schematically shown in the following reaction

(2) $$2CH_{3}OH\overset{E}{\to }CH_{3}OH_{2}^{+}+CH_{3}O^{-}\;,$$

which gives rise to the methanol cation methyloxonium CH${}_{3}$OH${}_{2}^{+}$ and to the anion methoxide CH${}_{3}$O${}^{-}$. There exist several interatomic distances capable to trace the molecular dissociation and the proton transfer of the above-mentioned chemical reactions. Among them, one of the most elegant quantities capable to monitor proton excursion events—and hence the dissociation degree of H-bonded systems—is the proton sharing coordinate. It is defined as $\delta =dOH-dO^{\prime }H$, where dOH is the covalent bond length of a reference molecule, whereas $dO^{\prime }H$ represents the length of the H-bond(s) the reference molecule donates, as schematically depicted in the insets of Figure 3. Of course, when the proton is transiently closer to the acceptor than to the donor oxygen, then δ>0. This variable has been successfully employed for investigating proton excursion events in liquid water simulated by means of different AIMD approaches and under standard conditions [64,65]. In Figure 3, the probability distributions of the proton sharing coordinate between methanol molecules (Figure 3a) and between water molecules (Figure 3b) of all the investigated systems are shown for a field strength of 0.30 V/Å. Albeit in the neat methanol and water samples (solid black curves) only very tiny fractions of molecules are partially dissociated at such a field strength, the situation drastically changes when methanol-water mixtures are concerned. In fact, for all the methanol-water mutual molar fractions investigated (i.e., 75:25, 50:50, and 25:75), the systems explore broader ranges of δ, both when the latter is defined for pairs of methanol molecules (Figure 3a) both when it is evaluated for pairs of water molecules (Figure 3b). As a consequence, conspicuously larger fractions of molecules are dissociated at 0.30 V/Å in the methanol-water mixtures with respect to their neat samples counterparts. In other words, mixing slightly lowers the molecular dissociation threshold, both for methanol and for water.

**Figure 4:**
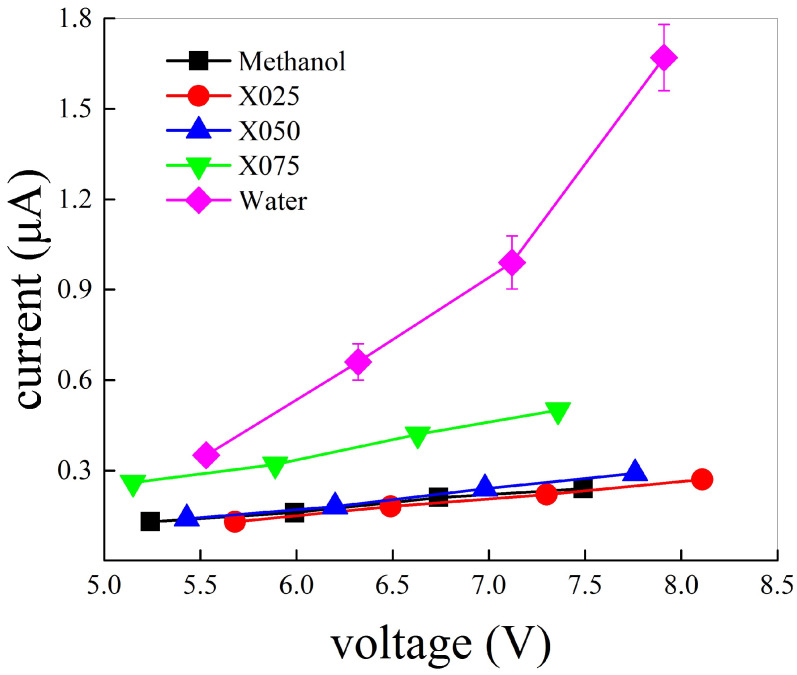
Protonic current-voltage diagram of neat methanol (black squares), methanol-water mixtures with molar ratios equal to 75:25 (red circles), 50:50 (upward blue triangles), 25:75 (downward green triangles), and neat water (magenta diamonds). Error bars are proportional to the number of conducting protons and can be discerned from the respective points only in the neat water case.

Once pairs of cations and anions of methanol and water species are formed, protons, under the action of external electric fields, are able to migrate across the H-bond networks via the well-known Grotthuss mechanism. In fact, starting from a field intensity of 0.35 V/Å, a protonic current has been detected in all the investigated systems. By counting the number of protons crossing an ideal plane placed at half of the simulations boxes’ edges and orthogonal to the field direction, a net proton flow can be measured. This way, the proton conductivity can be determined as:

(3) $$\sigma_{p}=\left(\frac{q\cdot \Delta N}{a^{2}\cdot \Delta t}\right)\cdot \frac{1}{E}\;\;,$$

where *q* is the elementary charge, *a* is the size of the box edge, ΔN is the number of protons crossing the surface area $a^{2}$ during the time interval Δt, and *E* is the field intensity. As shown by the current-voltage diagrams of Figure 4, all systems exhibit an Ohmic protonic response to the external field, provided that a conduction regime has been achieved. In spite of this similarity, conduction properties are very diversified among the investigated systems. Correlated proton hoppings across the H-bond network of the neat methanol sample lead to an ionic conductivity $\sigma_{p}\left(methanol\right)=0.2$ S·cm${}^{-1}$. This latter value is a half of the protonic conductivity recorded in a seminal work by some of us performed by employing Car-Parrinello MD (i.e., 0.4 S·cm${}^{-1}$) [28]. Such a discrepancy can be interpreted on a twofold basis. Firstly, the rate at which the electric field intensity has been raised in the current work is sizably lower than that of that work (i.e., 0.005 V·Å${}^{-1}$·ps${}^{-1}$ vs. 0.025 V·Å${}^{-1}$·ps${}^{-1}$). Albeit in both cases the simulations are clearly out of equilibrium, the present simulations allow to the H-bond networks to almost completely relax at each electric field strength explored. Typical relaxation times are indeed on the order of ∼3 ps in presence of the external fields beyond the dissociation threshold [28]. Secondly, crucially relevant non-local dispersion corrections have been taken into account in the current work. It is well-known indeed that dispersion-corrected GGA functionals are essential in describing H-bonds and the behavior of ionic species on them [66,67,68].

When relatively small amounts of water are added into methanol, no significant differences are induced in the capabilities of transferring protons. In fact, the same ionic conductivity measured in neat methanol is also recorded in the 75:25 and 50:50 methanol-water mixtures (i.e., 0.2 S·cm${}^{-1}$). However, when the amount of water exceeds the amount of methanol, an abrupt change of the conductivity properties is recorded. Although the ionic Ohmic behavior is preserved, the 25:75 methanol-water mixture exhibits a far larger conductivity with respect to the remainder samples with different molar ratios, as shown in Figure 4. In particular, a proton conductivity $\sigma_{p}\left(X075\right)=0.4$ S·cm${}^{-1}$ is recorded. This sudden increase of the conductivity is likely due to a structural change of the H-bond network, the latter representing the *locus* where correlated proton transfers take place. There exist experimental evidences that, at this specific concentration (i.e., 25:75), segregation of the water and methanol phases occurs [6], in that a bi-perculating H-bond network is established for such a molar ratio both in methanol-water mixtures [6] and in ethanol-water mixtures [7]. Hence, because of the more percolated H-bond network in water, exhibiting two donor and two acceptor sites of protons per molecule, a twice ionic conductivity is established with respect to the neat methanol case and the 75:25 and 50:50 methanol-water mixtures. The same rationale, corroborated by the fact that water molecules hold a larger number of protons *a priori* available for conduction, is capable to account for the noticeably larger proton conductivity measured in neat water. From the respective current-voltage diagram shown in Figure 4 (magenta diamonds), an ionic conductivity $\sigma_{p}\left(water\right)=1.3$ S·cm${}^{-1}$ is inferred. This value is about six times lower than that determined by means of pioneering Car-Parrinello MD simulations [29]. This implies, once again, that a richer statistics and dispersion-corrected GGA functionals are crucially important in describing water and the behavior of its ions [66,67,68]. In summary, under the action of intense static electric fields, the protonic subsystem of water is so far more conductive than that of methanol. Thus, when the amount of water exceeds that of methanol in methanol-water mixtures, proton conduction is sizably enhanced.

### 3.2. Field-Induced Chemical Reactions

Application of intense static electric fields on matter brings about a range of chemical effects which go well beyond the proton transfer reactions described up to now. In fact, electric fields are able to strongly influence redox- and electron-transfer reactions [69,70], to affect covalent and intermolecular bonds [71,72,73,74], to produce the well-known Stark effect [75] as well as the so-called vibrational Stark effect [76,77]. Besides, the field-enhanced chemical reactivity of neat methanol has been described only a few years ago [36,37]. However, the effects produced by intense electric fields on methanol-water mixtures, have not been reported so far.

As previously described by some of ours [36], field strengths on the order of 0.50 V/Å are able to trigger disproportionation reactions in neat methanol. This way, methanol molecules (CH${}_{3}$OH) are simultaneously reduced and oxidized leading to the formation of methane (CH${}_{4}$) and formaldehyde (H${}_{2}$CO), respectively, with the release of water (H${}_{2}$O) [36]. On the other hand, an additional reaction channel—pointing toward the formaldehyde formation—has been unveiled in the current work. In fact, for a field strength equal to 0.40 V/Å applied in neat methanol, the formation of formaldehyde and molecular hydrogen (H${}_{2}$) has been detected, as nominally laid out in the following reaction

(4) $$CH_{3}OH\overset{E}{\to }H_{2}CO+H_{2}\;\;.$$

**Figure 5:**
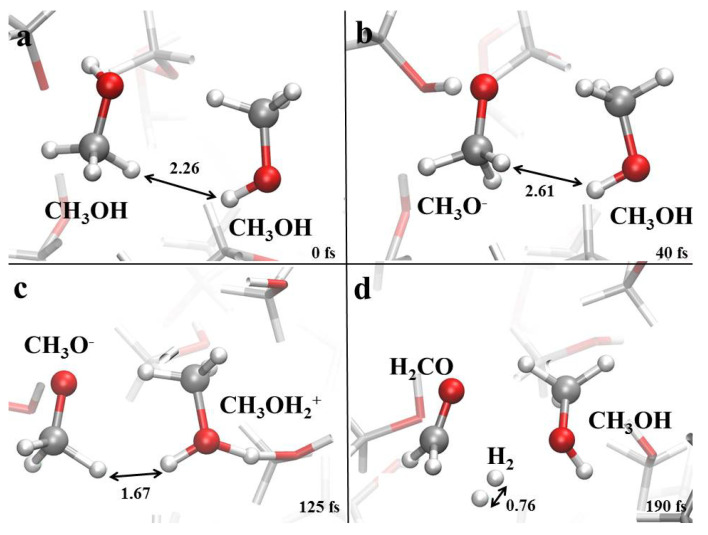
(**a**–**d**) Formaldehyde and molecular hydrogen formation mechanism in neat methanol and in a 75:25 methanol-water mixture under the action of a static electric field having intensity equal to 0.40 V/Å and 0.50 V/Å, respectively. White, red, and silver coloring refer to hydrogen, oxygen, and carbon atoms, respectively. A hydrogen-hydrogen distance, shown in Å, is highlighted to better follow the evolution of the reaction.

Of course, reaction (4) represents only a schematic representation of what is going on at a molecular level, where more complex interactions between the molecules take place. As shown in Figure 5a,c, methanol molecules strongly interact with the local environment under the action of the external field, in that they are able to rapidly exchange protons in the liquid. In this way, two first-neighboring neutral methanol molecules can evolve into methoxide (CH${}_{3}$O${}^{-}$) and methyloxonium (CH${}_{3}$OH${}_{2}^{+}$) in a hundreds of femtoseconds (fs) when a field intensity of 0.40 V/Å is applied. The molecular arrangement shown in Figure 5c suddenly leads to a recombination of the two counterions which, however, does not follow the canonical pathway involving a simple proton transfer. In fact, the closeness of two adjacent hydrogen atoms (Figure 5c), one belonging to the methyl group of CH${}_{3}$O${}^{-}$ and one to the positively charged head of the CH${}_{3}$OH${}_{2}^{+}$ cation, strongly perturbs the local electron densities. This leads to the prompt release of the highly reactive species hydride (H${}^{-}$) from methoxide and a proton (H${}^{+}$) from methyloxonium. Fast neutralization of the latter counterions culminates in the formation of formaldehyde and molecular hydrogen, with the consequent release of a methanol molecule, as shown in Figure 5d. The same mechanism has also been detected to occur in the 75:25 methanol-water mixture. Incidentally, synthesis of molecular hydrogen has recently been reported also for liquid neat ethanol exposed to static electric fields [59].

Notwithstanding the relevance of the reaction pathways connecting CH${}_{3}$OH to H${}_{2}$CO via the formation of CH${}_{4}$, on the one hand, and of H${}_{2}$, on the other, another reaction route governs the electrochemical response of liquid methanol to strong electric fields. At larger field strengths, the reaction network of the simplest alcohol exhibits, indeed, an accumulation basin where all chemical reactions point toward. In particular, the dehydration reaction leading to the synthesis of dimethyl ether (DME, CH${}_{3}$OCH${}_{3}$) has been observed, in agreement with previous simulations [37] and as schematically shown in the following

(5) $$2CH_{3}OH\overset{E}{\to }CH_{3}OCH_{3}+H_{2}O\;\;.$$

**Figure 6:**
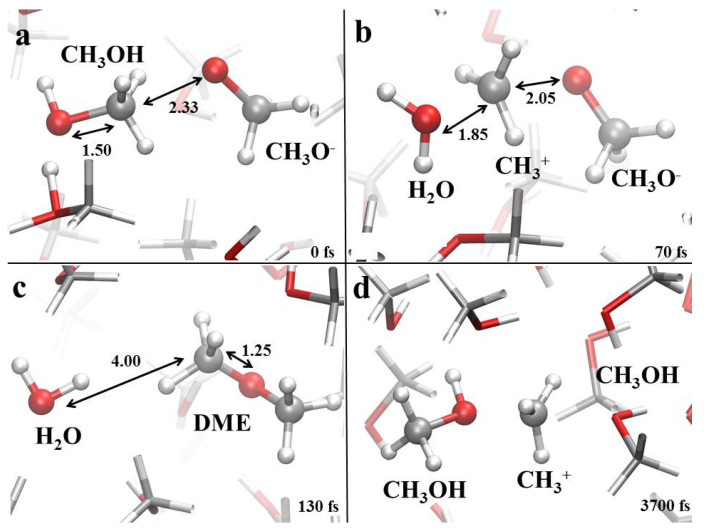
(**a**–**c**) Dimethyl ether (DME) formation mechanism in neat methanol, in the 75:25 and 50:50 methanol-water mixtures, under the action of a static electric field having intensity equal to 0.45 V/Å. (**d**) DME diffusion process through a methenium transfer mechanism. White, red, and silver coloring refer to hydrogen, oxygen, and carbon atoms, respectively. Some interatomic distances, shown in Å, are highlighted to better follow the progress of the reaction.

In this way, for fields as intense as 0.45 V/Å the reaction mechanism reported in Figure 6 has been observed not only in neat methanol but also in the 75:25 and 50:50 methanol-water mixtures. Besides, in this latter sample, such a reaction represents the unique chemical transformation recorded. Specific local molecular arrangements of methanol molecules (Figure 6a) and methanol counterions (Figure 6b), also in combination with the field direction [36,37], can lead to the cleavage of robust CO covalent bonds of the methyloxonium cation. As a consequence, methenium cations (CH${}_{3}^{+}$) are released–simultaneously to water molecules–and they recombine with adjacent methoxide anions (Figure 6b). Such a molecular process is quite fast and in about a hundreds of fs leads to the formation of water and DME (Figure 6c). It is noteworthy pointing out that, in some cases, DME behaves as a sort of defect in the neat methanol sample. In fact, it is able to migrate across the system not only by means of standard diffusion processes but also via correlated further chemical reactions where CH${}_{3}^{+}$ is transferred to adjacent methanol or methoxide molecules, as shown in Figure 6d. Such an ionic transfer process, resembling that of proton transfer, leads to a subsequent DME formation with the release of a methanol molecule.

As stated before, an important reaction is represented by the one-pot synthesis of formaldehyde and methane with the release of water, as nominally shown in the following

(6) $$2CH_{3}OH\overset{E}{\to }H_{2}CO+CH_{4}+H_{2}O.$$

**Figure 7:**
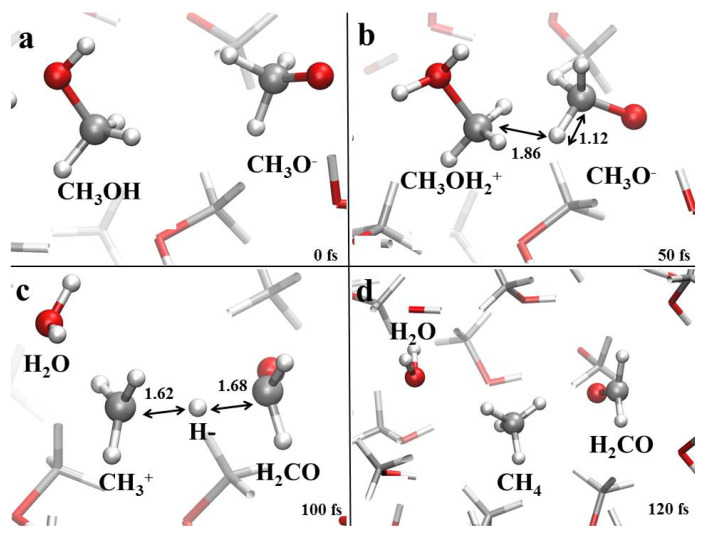
(**a**–**d**) Formaldehyde and methane formation mechanism in neat methanol and in a 75:25 methanol-water mixture under the action of a static electric field having intensity equal to 0.50 V/Å.

Such a reaction has already been investigated in neat methanol by some of ours [36]. On the other hand, it does not take place uniquely in pure methanol. In fact, we have observed it also in the 75:25 methanol-water mixture. However, as shown in Figure 7a, it strictly requires the intervention of (and the tight interaction between) two methanol molecules which evolve into the methanol counterions (Figure 7b). This means that the larger the initial amount of water the lower the probability of finding two adjacent methanol molecules. As a consequence, similarly to the molecular hydrogen-yielding process of Figure 5, the reaction depicted in Figure 7 and leading to the synthesis of formaldehyde, methane, and water has been observed in neat methanol and in the 75:25 methanol-water mixture only.

**Figure 8:**
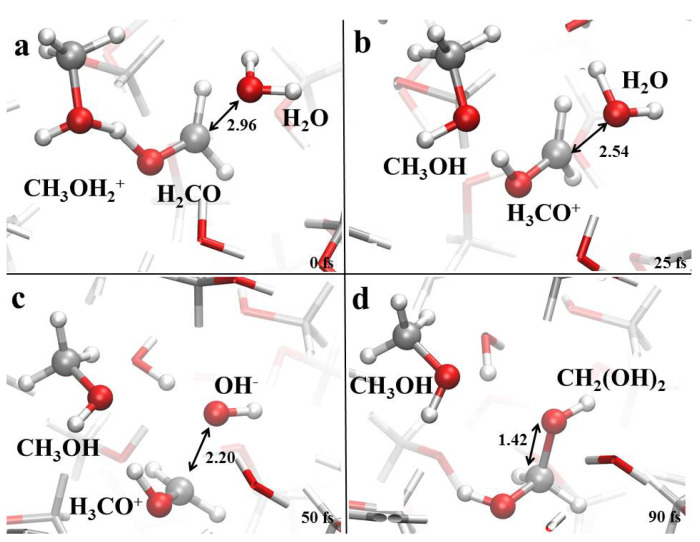
(**a**–**d**) Formaldehyde monohydrate formation mechanism in neat methanol and in a 75:25 methanol-water mixture under the action of a static electric field of 0.50 V/Å.

Finally, once formaldehyde is formed in some of the investigated samples (i.e., in neat methanol and in the 75:25 methanol-water mixture) it can react with the local environment, as shown in Figure 8. As a consequence of a proton transfer event (Figure 8a,b), formaldehyde may evolve into its cationic form H${}_{3}$CO${}^{+}$, which is able to attract adjacent hydroxide ions (Figure 8c) and hence lead to the formaldehyde monohydrate (CH${}_{2}$(OH)${}_{2}$) synthesis (Figure 8d).

Albeit under the action of the field samples such as neat methanol and the 75:25 methanol-water mixture undergo to a series of diversified chemical reactions, the 50:50 methanol-water mixture exhibits only a unique reaction pathway (i.e., that leading to the DME synthesis). Besides, the 25:75 methanol-water mixture and the neat water sample do not exhibit chemical reactions more complex than simple proton transfers, indicating that the presence of water strongly inhibits chemical reactivity. Such a finding can be interpreted not only on the basis that the presence of Carbon atoms drastically enhances the reactivity of methanol-containing samples, but also on the evidence that the vast majority of the presented reactions yields water as a by-product. This clearly shifts the equilibrium of the reactions toward the reactants in water-containing samples, as summarized in Table 1.

## 4. Conclusions

In this work, we have presented the results stemming from state-of-the-art ab initio molecular dynamics (AIMD) simulations conducted at room temperature and on a series of H-bonded liquids exposed to external static electric fields. In particular, the physical and chemical effects induced by those fields on neat methanol, neat water and methanol-water mixtures at different molar ratios (i.e., 75:25, 50:50, and 25:75) have been described. It turned out that the mixing of methanol and water increases the proton excursion events with respect to their counterparts in neat methanol and in pure water. This leads to a slight lowering of the field-induced molecular dissociation threshold in the mixed phases.

Besides, proton conduction properties drastically depends on the amount of water solvated in methanol. Whereas neat methanol, the 75:25 and the 50:50 methanol-water mixtures exhibit the same proton conductivity (i.e., 0.2 S·cm${}^{-1}$), a sizable increase is recorded in the 25:75 methanol-water mixture, the latter being characterized by a twice ionic conductivity (i.e., 0.4 S·cm${}^{-1}$). Consistently with other findings, such an evidence may be ascribed to a sudden structural change of the H-bond network. As expected, a noticeably larger protonic conductivity equal to 1.3 S·cm${}^{-1}$ has been recorded in the neat water sample, indicating that proton transfers take place more easily in more percolated H-bond networks.

By contrast, the presence of water in methanol strongly inhibits the catalytic properties carried by the external electric field. If, on the one hand, under the intense field regime (i.e., beyond 0.40 V/Å) a multifaceted reaction network is observed in neat methanol and in the 75:25 methanol-water mixture where synthesis of e.g., hydrogen, dimethyl ether, formaldehyde and methane is afforded, on the other, only one reaction is recorded in the 50:50 methanol-water mixture. Moreover, no chemical reactions more complex than simple proton transfers have been detected in the 25:75 methanol-water mixture and in the neat water sample. This is mainly due to the fact that the application of static fields in methanol and methanol-water mixtures preferentially induces dehydration reactions (i.e., water is released as a by-product), pushing the chemical equilibrium in water-containing samples toward the reactants. This way, the presence of water strongly hampers the chemical reactivity of methanol.

## Figures and Tables

**Table 1:** Percent fractions of the molecular species present in the systems at the end of each simulation at 0.50 V/Å and generated by the field. Percentages are determined as the ratio between the number of formed molecules of a given species and the number of methanol molecules composing the original sample.

Molecular Species (%)	Methanol	75:25 mix	50:50 mix	25:75 mix	Water
Formaldehyde	4	2	−	−	−
Methane	2	2	−	−	−
Hydrogen	2	2	−	−	−
Water	6	5	3	−	−
Formaldehyde monohydrate	−	2	−	−	−
Dimethyl ether (DME)	6	2	3	−	−
